# Influence of Reader Expertise on Myocardial Infarction Detection: A Comparative Study of Dark-Blood and Bright-Blood Late Gadolinium Enhancement MRI

**DOI:** 10.1097/RLI.0000000000001161

**Published:** 2025-02-24

**Authors:** Bibi Martens, Lara R. van der Meulen, Richard J. Crawley, Yvonne J.M. van Cauteren, Martijn W. Smulders, Sebastian Streukens, Babs M.F. Hendriks, Ivo P.L. Houben, Suzanne Gommers, Simon M. Frey, Lloyd Brandts, Joachim E. Wildberger, Amedeo Chiribiri, Robert J. Holtackers

**Affiliations:** Department of Radiology and Nuclear Medicine, Maastricht University Medical Centre, Maastricht, the Netherlands (B.M., L.R.v.d.M., Y.J.M.v.C., B.M.F.H., S.G., J.E.W., R.J.H.); Research Institute for Oncology and Reproduction (GROW), Maastricht University, the Netherlands (B.M.); Cardiovascular Research Institute Maastricht (CARIM), Maastricht University, the Netherlands (L.R.v.d.M., Y.J.M.v.C., M.W.S., B.M.F.H., J.E.W., R.J.H.); School of Biomedical Engineering and Imaging Sciences, King's College London, St Thomas' Hospital, London, United Kingdom (R.J.C., S.M.F., A.C., R.J.H.); Department of Cardiology, Maastricht University Medical Centre, Maastricht, the Netherlands (Y.J.M.v.C., M.W.S., S.S.); Department of Radiology and Nuclear Medicine, Zuyderland, Heerlen, the Netherlands (I.P.L.H.); Department of Cardiology, University Hospital Basel, Basel, Switzerland (S.M.F.); and Department of Clinical Epidemiology and Medical Technology Assessment, Maastricht University Medical Centre, Maastricht, the Netherlands (L.B.)

**Keywords:** myocardial infarction, magnetic resonance imaging, late gadolinium enhancement, bright-blood LGE, dark-blood LGE, expertise, reader confidence

## Abstract

**Objectives:**

This study aimed to evaluate the influence of reader training and experience on the detection of (small) myocardial infarctions (MIs) and the assessment of ischemic scar transmurality using dark-blood late gadolinium enhancement (LGE) and bright-blood LGE magnetic resonance imaging. It was hypothesized that dark-blood LGE simplifies the detection of (small) MIs for less experienced readers, compared with bright-blood LGE imaging.

**Materials and Methods:**

One hundred patients referred for cardiac magnetic resonance imaging for suspected ischemic scar were retrospectively included. Dark-blood LGE was performed first, followed by bright-blood LGE. Nine clinicians, grouped into three levels based on their training and experience, assessed the LGE images for the presence of MI and ischemic scar transmurality. Their assessment was subsequently compared with a European Association of Cardiovascular Imaging level 3 consultant. Reader confidence was evaluated with a 4-point Likert scale. Multilevel logistic regression was used to compare the 2 LGE methods and assess differences in myocardial infarction detection and transmurality among the 3 experience levels. Wilcoxon signed rank tests were performed to compare the reader confidence between the 2 LGE methods, whereas Friedman omnibus tests were conducted to assess differences in reader confidence among the 3 experience levels.

**Results:**

Dark-blood LGE resulted in increased correct detection of MIs compared with bright-blood LGE for both level 1 (87.3% vs 82.7%, odds ratio [OR]: 1.55 [95% confidence interval (CI): 0.94–2.54], *P* = 0.083) and level 2 readers (89.7% vs 83.0%, OR: 2.05 [95% CI: 1.20–3.51], *P* = 0.009). There was no significant difference observed between dark-blood LGE and bright-blood LGE for level 3 readers (88.7% vs 87.0%, OR: 1.20 [95% CI: 0.70–2.06], *P* = 0.495). Level 2 readers significantly detected more small MIs correctly when using dark-blood LGE compared with bright-blood LGE (66.7% vs 50.8%, OR: 2.40 [95% CI: 1.03–5.60], *P* = 0.042). All experience levels showed significantly increased confidence in presence of ischemic scar and transmurality with dark-blood LGE.

**Conclusions:**

Readily available dark-blood LGE can assist less experienced readers in correctly detecting and assessing (small) MIs compared with conventional bright-blood LGE. Regardless of experience level, dark-blood LGE improves reader confidence in assessing the presence and transmurality of MIs.

Over the past 2 decades, trends for prevalence and mortality of ischemic heart disease (IHD) have been decreasing.^1^ However, IHD continues to be the leading cause of death worldwide in both men and women.^2^–^4^ Myocardial infarction (MI) is a common presentation of IHD, and the myocardial scar tissue can be detected using late gadolinium enhancement (LGE) magnetic resonance imaging (MRI).^5^ It has been shown that even small regions of myocardial scar tissue can increase the risk of major adverse cardiac events by 7-fold.^6^,^7^ In addition, accurately assessing the transmural extent of ischemic scar regions is equally important as it proved to be a major predictor of successful functional recovery after revascularization.^8^

In conventional bright-blood LGE, the blood pool appears bright due to the nulling of the myocardium, making it challenging to distinguish between the blood pool and scar tissue, which often appears equally bright.^9^ An easy-to-use and readily available “dark-blood,” more specifically “gray-blood,” LGE method has been proposed to overcome the poor scar-to-blood contrast.^9^ Previous studies have demonstrated that this dark-blood LGE method is superior in detecting MIs.^9^,^10^

Both conventional bright-blood and dark-blood LGE images are generally evaluated through visual qualitative assessment only. In this regard, the Society for Cardiovascular Magnetic Resonance states that “readers should have adequate training and clinical experience” when assessing cardiac MRI scans.^11^ A study by Nies et al^12^ showed that a less experienced reader was more confident when using reconstructed 2D dark-blood LGE compared with 2D bright-blood LGE for the detection of ischemic scar. Research evaluating different LGE methods often uses only experts to review the images.^13^,^14^ However, in clinical practice, less experienced readers may also review the LGE images, possibly influencing the patients' outcomes. To the best of our knowledge, no studies have researched the impact of the level of training and experience of readers on detecting MIs using different LGE MRI methods.

Therefore, the present study aims to determine the influence of reader training and experience on the assessment of (*a*) (small) MIs, (*b*) ischemic scar transmurality, and (*c*) reader confidence in detecting the ischemic scar tissue and its transmurality using both dark-blood and bright-blood LGE MRI. It is hypothesized that dark-blood LGE cardiac MRI improves the detection of (small) MIs, especially for less experienced readers, compared with bright-blood LGE imaging.

## MATERIALS AND METHODS

### Study Population

A total of 100 patients referred for cardiac MRI for suspected ischemic scar were retrospectively included. Patients were excluded from the study if they had suspected cardiomyopathy, a history of percutaneous coronary intervention and/or coronary artery bypass graft, and a cardiac device. Additional exclusion criteria included general contraindications to MRI, such as gadolinium allergy and claustrophobia. The study was approved by the North of Scotland Research Ethics Committee (15/NS/0030), and all participants provided written informed consent for their participation and use of data for research purposes. The study was conducted according to the principles of the Declaration of Helsinki.

### MRI Protocol

All patients underwent cardiac MRI examination using a whole-body clinical 3 T MRI scanner (Achieva TX; Philips Healthcare, Best, the Netherlands) and a 32-channel cardiac phased-array coil. Standard cine imaging was performed in the short axis, 2-chamber, 4-chamber, and left ventricular outflow tract views for volumetric and functional assessment. Approximately 10 minutes after intravenous injection of 0.2 mmol/kg gadobutrol (Gadovist; Bayer Pharmaceuticals, Berlin, Germany), dark-blood LGE was performed first, followed by conventional bright-blood LGE, as per the hospital's standard clinical protocol. Both LGE methods were performed in the same 3 long-axis views and stack of short-axis views as employed for cine imaging. For both LGE methods, an identical standard segmented 2D phase-sensitive inversion-recovery (PSIR) LGE sequence was used, except from the inversion time (TI) parameter, which was set differently. This TI was set to null the blood pool of the left ventricle for dark-blood LGE (making it appear gray in the PSIR reconstruction), while it was set to null the normal myocardium for conventional bright-blood LGE. TIs were individually selected based on separate Look-Locker sequences performed prior to both LGE acquisitions. All LGE images were acquired in mid-diastole during respiratory breath-holding. The administered contrast dose adheres to local protocol and current international guidelines.^5^

### Reader Selection

A total of 9 clinicians, both radiologists and cardiologists, were selected from 4 European hospitals and grouped into 3 levels (3 readers for each level) based on their competency, as outlined by the European Association of Cardiovascular Imaging (EACVI) guidelines.^15^ While the EACVI certification was not a mandatory requirement for selection, readers were assigned to the different levels according to these criteria. Level 1 readers had either completed the EACVI level 1 course and/or reviewed at least 50 cardiac MRI cases within the 12 months prior to the study, in addition to earning 20 continuing medical education (CME) hours in cardiovascular imaging. Level 2 readers were required to either have EACVI level 2 certification and/or reviewed at least 150 cardiac MRI cases within the same 12-month period, earned 50 CME hours, and completed a minimum of 3 months of training in an accredited center. Finally, level 3 readers had either completed EACVI level 3 certification and/or reviewed at least 300 cardiac MRI cases in the 12 months preceding the study, earned 50 CME hours, and completed at least 12 months of training in an accredited center.^16^

### Image Analysis

All imaging data were randomly divided into 2 sets. Set 1 consisted of 100 scans including the cine images with either dark-blood or bright-blood LGE images of each patient, whereas set 2 consisted of the cine images and the complimentary set of dark-blood or bright-blood LGE images of each patient. This ensured that each reader reviewed the images of only 1 LGE method per patient in the first set of 100 scans (either dark-blood LGE or bright-blood LGE). After an interval of at least 2 weeks, the other complimentary set of 100 scans was presented to the same reader. All readers were blinded to the patients' clinical information and prior reports.

Using the 16-segment heart model, the readers assessed each segment for the presence of potentially viable infarction (≤50% LGE transmurality), nonviable infarction (>50% LGE transmurality), or no infarction.^17^,^18^ Small infarctions were defined as involving 1 or 2 segments, whereas large infarctions were defined as involving 3 or more segments. These outcomes were compared with the findings described in the original patient report, which served as the reference standard for the study. The report was evaluated and approved by a single EACVI level 3 consultant, not included as a reader in the study, who will be further referred to as the “expert.” In addition, readers stated their confidence in both the presence of ischemic scar and ischemic scar transmurality on a 4-point Likert scale (0 = not confident, 1 = somewhat confident, 2 = confident, and 3 = very confident).

**TABLE 1:** Baseline Characteristics of the Study Population (n = 100)

	n = 100
Age in years, median (range)	63.5 (26–89)
Women, n (%)	28 (28.0)
Weight in kg, median (IQR)	86.0 (72.3–96.0)
Height in cm, median (IQR)	170.0 (163.3–178.0)
BMI in kg/m^2^, median (IQR)	28.4 (25.7–32.8)
Infarction, n (%)	75 (75.0)
No. segments infarcted, median (IQR)	3.0 (0.3–5.0)
Size of infarction, n (%)	
No infarction	25 (25.0)
Small infarction	21 (21.0)
Large infarction	54 (54.0)
Transmurality, n (%)	
No infarction	25 (25.0)
Viable infarction	53 (53.0)
Nonviable infarction	22 (22.0)

n, number of patients; IQR, interquartile range; BMI, body mass index.

### Statistical Analysis

Results are presented as mean ± standard deviation (SD) for normally distributed data, median with interquartile range (IQR) for nonnormally distributed data, or as a percentage, unless stated otherwise. Differences in the rates of correctly identified infarctions between dark-blood LGE and bright-blood LGE were tested using a multilevel binary logistic regression model, which accounted for the hierarchical structure of the data (ie, multiple readers assessing multiple patients). The dependent variable in the regression model was correctly identified infarction and the independent variable (fixed effect) was the LGE method. An interaction between the LGE method and size was tested. Random intercepts for both patients and readers were included. Odds ratios (ORs) with 95% confidence intervals (CIs) were reported for the fixed effects. The 3 levels of reader experience were compared using a multilevel binary logistic regression model, similar to the one previously described, with the key difference that the independent variable (fixed effect) was the experience level. The analysis of reader confidence was limited to cases in which the reader identified the presence of an infarction. Differences in reader confidence between dark-blood LGE and bright-blood LGE were tested using a paired *t* test (for normally distributed data) or the Wilcoxon signed rank test (for nonnormally distributed data). Additionally, reader confidence across the 3 levels of reader experience was compared using 1-way analysis of variance with repeated measures with post hoc pairwise comparisons or the Friedman omnibus test with Wilcoxon signed rank post hoc test, depending on distribution of the data. Normality of data was assessed using the Kolmogorov-Smirnov test, and Bonferroni correction was used to correct for multiple testing. To assess the interreader reliability, intraclass correlation coefficients (ICCs) were calculated for each level and LGE method based on a mean-rating (k = 3), absolute-agreement, 2-way mixed-effects model. All statistical analyses were 2-tailed and performed using SPSS (version 27; IBM, Armonk, NY) with a significance level of 5%.

## RESULTS

### Study Population

**FIGURE 1:**
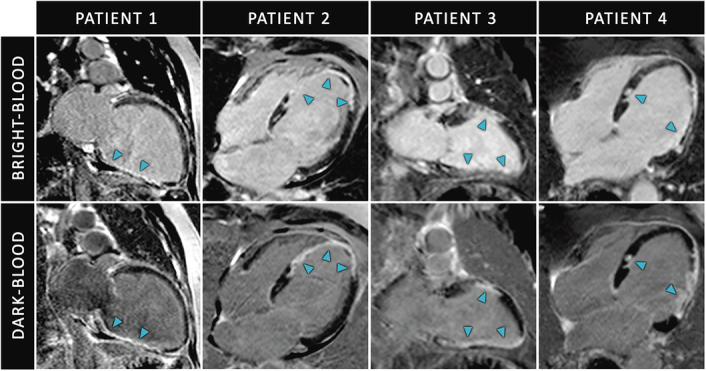
Conventional bright-blood and dark-blood late gadolinium enhancement (LGE) cardiac magnetic resonance images. The blue arrowheads indicate ischemic scars.

The baseline characteristics of the study population are summarized in Table 1. Among the included patients (n = 100), 28 were women, and the median age was 63.5 (range, 26–89) years. Based on expert consensus, the prevalence of MI in this patient cohort was 75% (75/100). In these 75 patients with MI, 54 patients (72.0%) had a large infarction (≥3 segments involved), whereas 21 patients (28.0%) had a small infarction (1 or 2 segments involved). Fifty-three (70.7%) infarctions were viable, and 22 (29.3%) infarctions were nonviable. Imaging examples of both LGE methods can be found in Figure 1.

### Correct Identification of Myocardial Infarction

**FIGURE 2:**
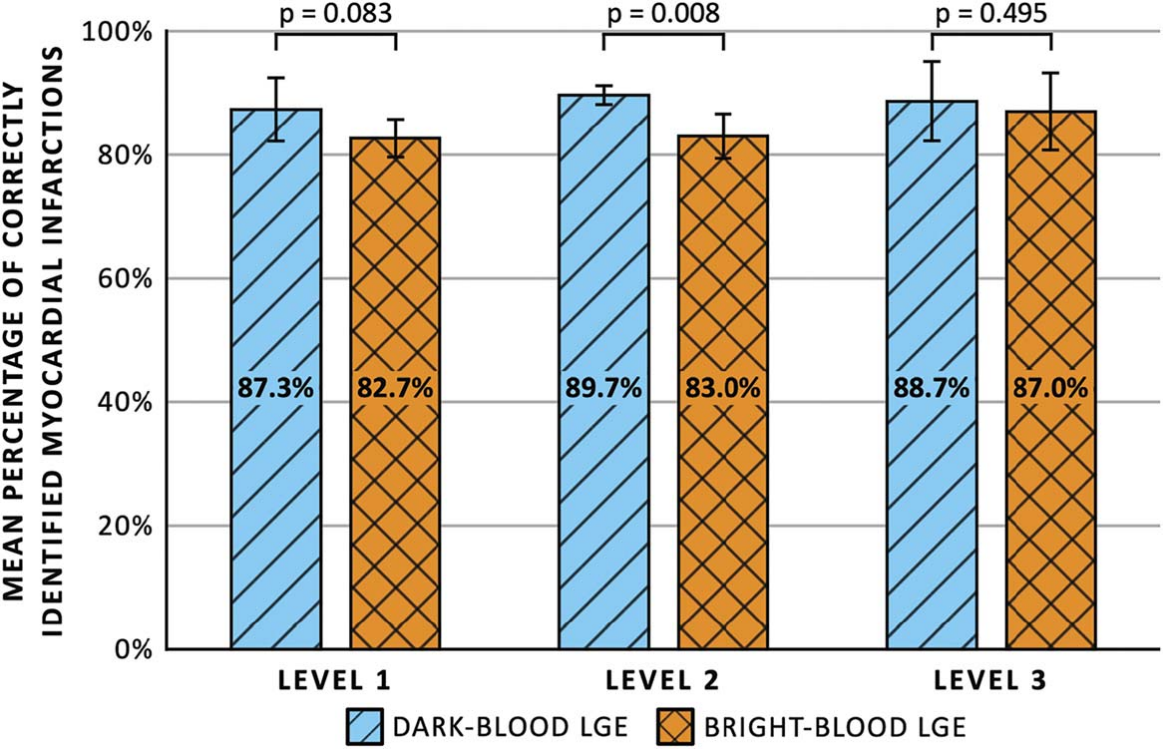
Mean percentage of correctly identified myocardial infarctions comparing between dark-blood late gadolinium enhancement (LGE) and bright-blood LGE for different levels of cardiovascular magnetic resonance training and experience.

**FIGURE 3:**
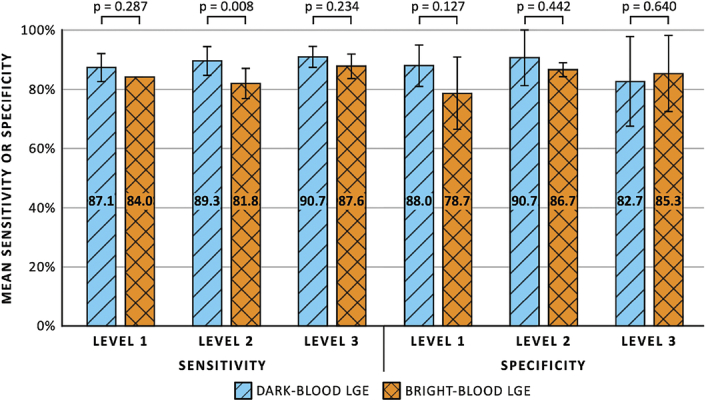
Mean sensitivity and specificity comparing between dark-blood late gadolinium enhancement (LGE) and bright-blood LGE for different levels of cardiovascular magnetic resonance training and experience.

Level 1 readers showed agreement with the expert for correct identification of MI in 87.3% ± 4.2% (262/300 assessments) and 82.7% ± 2.5% (248/300) of the cases when using dark-blood LGE and bright-blood LGE, respectively (Fig. 2). The percentages slightly increased for level 2 readers to 89.7% ± 1.5% (269/300) and 83.0% ± 3.6% (249/300), respectively. Finally, for level 3 readers, the agreement was 88.7% ± 6.4% (266/300) and 87.0% ± 6.2% (261/300), respectively. Supplemental Digital Content 1, http://links.lww.com/RLI/A1000, shows the percentage of correctly identified MIs for the individual readers. Odds ratios for correctly identifying MIs using dark-blood LGE compared with bright-blood LGE were 1.55 (95% CI: 0.94–2.54, *P* = 0.083) for level 1 readers, 2.05 (95% CI: 1.20–3.51, *P* = 0.008) for level 2 readers, and 1.21 (95% CI: 0.70–2.06, *P* = 0.495) for level 3 readers. There was good interreader reliability for the 3 readers within each level (ICC > 0.75 for all levels). No significant differences in the odds for correctly identifying MI were observed among the 3 levels of reader experience, using either dark-blood LGE or bright-blood LGE (Table 2). The sensitivity and specificity of dark-blood LGE and bright-blood LGE for the different levels of reader experience are shown in Figure 3. No significant differences in sensitivity and specificity were observed between the 2 LGE methods and among the 3 levels of reader experience, except that level 2 readers showed a significantly higher sensitivity when using dark-blood LGE compared with bright-blood LGE (89.3% ± 4.8% vs 81.8% ± 5.0%, OR: 2.46 [95% CI: 1.27–4.76], *P* = 0.008).

**TABLE 2:** Results of Multilevel Logistic Regression Analysis, Including Odds Ratios (OR), 95% Confidence Intervals (CI), and *P* Values, Comparing the Odds of Correctly Identifying Myocardial Infarctions Using Dark-Blood Late Gadolinium Enhancement (LGE) and Bright-Blood LGE Across Different Levels of Cardiovascular Magnetic Resonance Training and Experience

	OR	95% CI	*P*
Level 1			
Dark-blood LGE	1.55	0.94–2.54	0.083
Bright-blood LGE	Reference		
Level 2			
Dark-blood LGE	2.05	1.20–3.51	0.009
Bright-blood LGE	Reference		
Level 3			
Dark-blood LGE	1.21	0.70–2.06	0.495
Bright-blood LGE	Reference		
Dark-blood LGE			
Level 1	0.82	0.28–2.40	0.711
Level 2	1.07	0.36–3.18	0.904
Level 3	Reference		
Bright-blood LGE			
Level 1	0.62	0.28–1.38	0.240
Level 2	0.64	0.29–1.42	0.275
Level 3	Reference		

### Small Myocardial Infarction Identification

**FIGURE 4:**
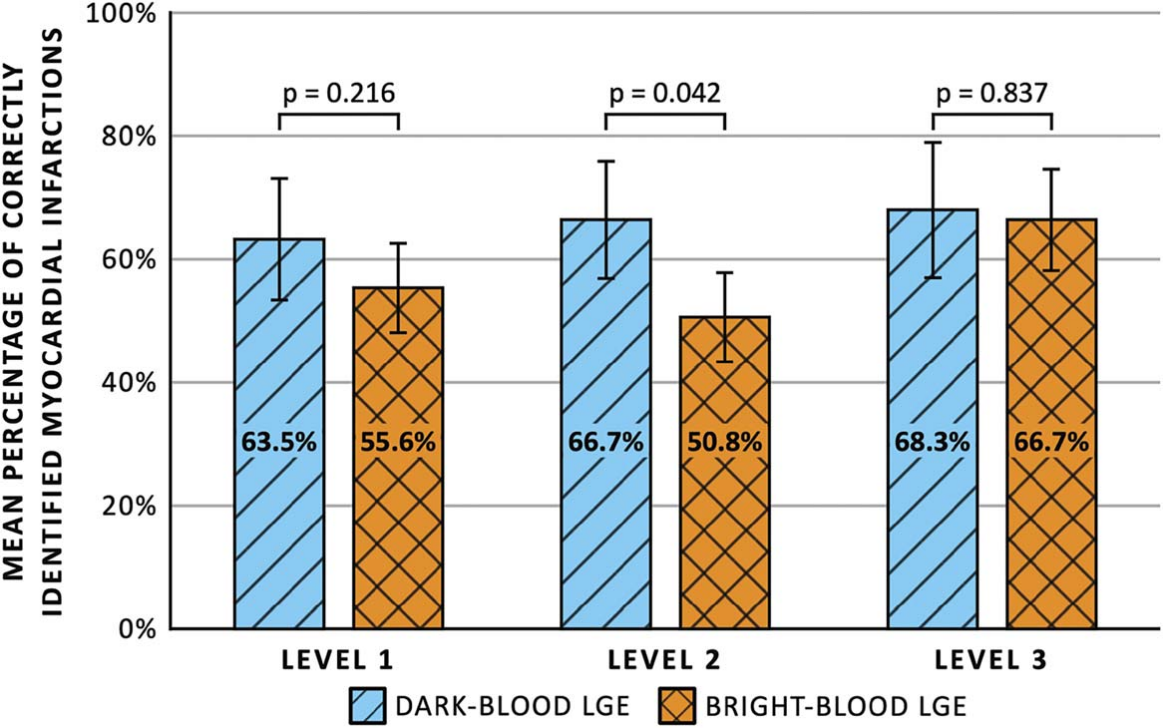
Mean percentage of correctly identified small myocardial infarctions comparing between dark-blood late gadolinium enhancement (LGE) and bright-blood LGE for different levels of cardiovascular magnetic resonance training and experience.

Level 1 readers were able to correctly identify 63.5% ± 9.9% (40/63 assessments) and 55.6% ± 7.3% (35/63) of the small MIs with dark-blood LGE and bright-blood LGE, respectively (Fig. 4). For level 2 readers, the rates of correctly identified small MIs were 66.7% ± 9.5% (42/63) and 50.8% ± 7.3% (32/63), respectively. Level 3 readers correctly identified 68.3% ± 11.0% (43/63) and 66.7% ± 8.2% (42/63) of MIs with dark-blood LGE and bright-blood LGE, respectively. Odds ratios for correctly identifying small MIs using dark-blood LGE compared with bright-blood LGE were 1.50 (95% CI: 0.67–3.36, *P* = 0.316) for level 1 readers, 2.40 (95% CI: 1.03–5.60, *P* = 0.042) for level 2 readers, and 1.09 (95% CI: 0.48–2.46, *P* = 0.837) for level 3 readers. Level 2 readers were significantly worse at identifying small MIs correctly compared with level 3 readers (50.8% vs 66.7%, OR: 0.41 [95% CI: 0.17–0.95], *P* = 0.038) when using bright-blood LGE. Apart from this, no significant differences were observed among the 3 levels of reader experience for either dark-blood LGE or bright-blood LGE. (Table 3).

**TABLE 3:** Results of Multilevel Logistic Regression Analysis, Including Odds Ratios (OR), 95% Confidence Intervals (CI), and *P* Values, Comparing the Odds of Correctly Identifying Small Myocardial Infarctions Using Dark-Blood Late Gadolinium Enhancement (LGE) and Bright-Blood LGE Across Different Levels of Cardiovascular Magnetic Resonance Training and Experience

	OR	95% CI	*P*
Level 1			
Dark-blood LGE	1.50	0.67–3.36	0.316
Bright-blood LGE	Reference		
Level 2			
Dark-blood LGE	2.40	1.03–5.60	0.042
Bright-blood LGE	Reference		
Level 3			
Dark-blood LGE	1.089	0.48–2.46	0.837
Bright-blood LGE	Reference		
Dark-blood LGE			
Level 1	0.73	0.24–2.18	0.568
Level 2	0.90	0.30–2.70	0.846
Level 3	Reference		
Bright-blood LGE			
Level 1	0.53	0.23–1.24	0.140
Level 2	0.41	0.17–0.95	0.038
Level 3	Reference		

### Transmurality of Myocardial Infarction

Among the infarctions as assessed by the expert, level 1 readers were able to correctly assess the transmurality of the MI in 58.7% ± 8.7% (132/225) and 56.4% ± 7.6% (127/225) of the cases with dark-blood LGE and bright-blood LGE, respectively. For level 2 readers, the rates of correctly assessing the transmurality of the MI slightly increased to 63.1% ± 5.4% (142/225) and 57.3% ± 4.6% (129/225), respectively. Level 3 readers correctly assessed the transmurality of the MI in 64.4% ± 7.7% (145/225) and 61.8% ± 3.4% (139/225) of the cases with dark-blood LGE and bright-blood LGE, respectively. Odds ratios for correctly assessing transmurality of MIs using dark-blood LGE compared with bright-blood LGE were 1.13 (95% CI: 0.73–1.75, *P* = 0.581) for level 1 readers, 1.38 (95% CI: 0.89–2.13, *P* = 0.151) for level 2 readers, and 1.19 (95% CI: 0.74–1.89, *P* = 0.476) for level 3 readers. For both dark-blood LGE and bright-blood LGE, no significant differences among the 3 levels of reader experience were observed (Table 4).

**TABLE 4:** Results of Multilevel Logistic Regression Analysis, Including Odds Ratios (OR), 95% Confidence Intervals (CI), and *P* Values, Comparing the Odds of Correctly Assessing the Transmurality of Myocardial Infarctions Using Dark-Blood Late Gadolinium Enhancement (LGE) and Bright-Blood LGE Across Different Levels of Cardiovascular Magnetic Resonance Training and Experience

	OR	95% CI	*P*
Level 1			
Dark-blood LGE	1.13	0.73–1.75	0.581
Bright-blood LGE	Reference		
Level 2			
Dark-blood LGE	1.38	0.89–2.13	0.151
Bright-blood LGE	Reference		
Level 3			
Dark-blood LGE	1.19	0.74–1.89	0.476
Bright-blood LGE	Reference		
Dark-blood LGE			
Level 1	0.68	0.36–1.29	0.236
Level 2	0.91	0.48–1.73	0.783
Level 3	Reference		
Bright-blood LGE			
Level 1	0.72	0.42–1.23	0.233
Level 2	0.76	0.45–1.30	0.318
Level 3	Reference		

### Reader Confidence

**FIGURE 5:**
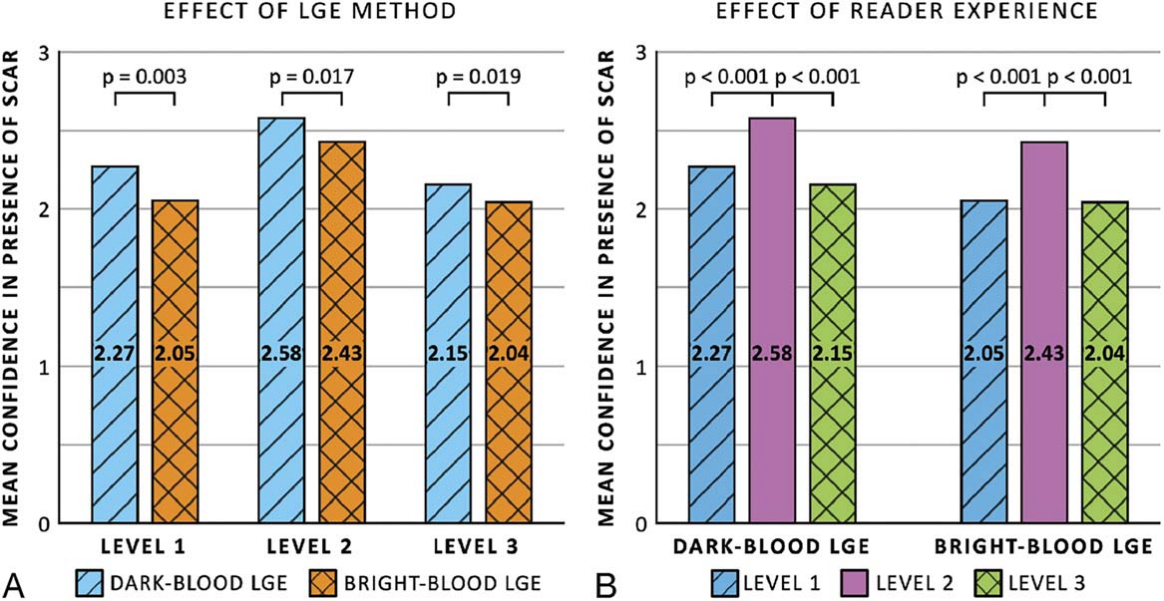
Mean confidence scores in presence of ischemic scar comparing between dark-blood late gadolinium enhancement (LGE) and bright-blood LGE (A) and among different levels of cardiovascular magnetic resonance training and experience (B).

Reader confidence in detecting the presence of ischemic scar tissue was significantly higher with dark-blood LGE compared with bright-blood LGE in all 3 experience levels (*P* = 0.003, *P* = 0.017, and *P* = 0.019, respectively) (Fig. 5A). Overall, level 2 readers were significantly more confident than level 1 and level 3 readers, both when using dark-blood LGE (*P* < 0.001 for both) and bright-blood LGE (*P* < 0.001 for both) (Fig. 5B).

**FIGURE 6:**
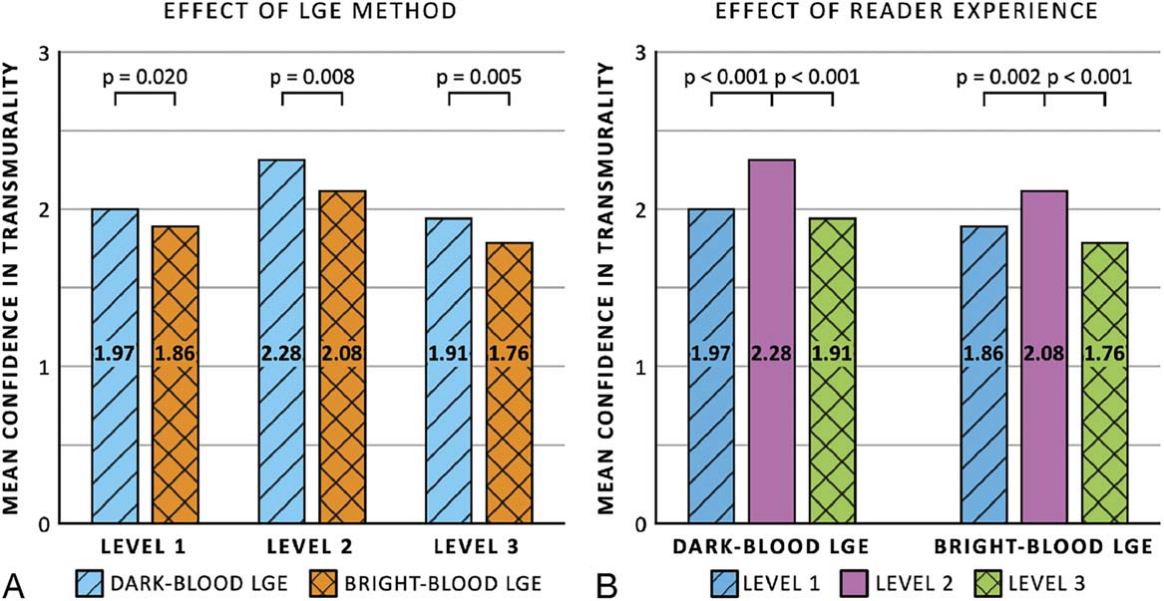
Mean confidence scores in presence of ischemic scar transmurality comparing between dark-blood late gadolinium enhancement (LGE) and bright-blood LGE (A) and among different levels of cardiovascular magnetic resonance training and experience (B).

For confidence regarding the transmurality of the ischemic scars, readers of all 3 experience levels showed significantly increased confidence when using dark-blood LGE compared with bright-blood LGE (*P* = 0.020, *P* = 0.008, and *P* = 0.005, respectively) (Fig. 6A). Level 2 readers were significantly more confident in assessing ischemic scar transmurality than level 1 and level 3 readers, both when using dark-blood LGE (*P* < 0.001 for both) and bright-blood LGE (*P* = 0.002 and *P* < 0.001) (Fig. 6B).

## DISCUSSION

This study determined the influence of the level of training and experience of cardiac MRI readers on the assessment of (small) MIs, ischemic scar transmurality, and on their confidence using dark-blood and bright-blood LGE imaging. Three key results of this study are as follows. First, when using dark-blood LGE, improved detection of MIs was observed compared with bright-blood LGE for level 1 and level 2 readers, with a significant difference for the latter group. For level 3 readers, no significant difference between dark-blood LGE and bright-blood LGE was observed. Second, when only considering small MIs, dark-blood LGE enabled level 1 and level 2 readers to detect more of these small MIs compared with conventional bright-blood LGE, with a significant difference for the latter group. For level 3 readers, both LGE methods performed similarly in detecting small MIs. Third, readers were more confident in assessing the presence of ischemic scar and ischemic scar transmurality when using dark-blood LGE. These findings are in line with the hypothesis that for experienced readers, the choice of LGE method is less critical, whereas less experienced readers benefit more from using dark-blood LGE when detecting MIs compared with using bright-blood LGE.

A recent study by Nies et al^12^ compared reader confidence of a single beginner and expert reader in detecting myocardial scar on cardiac MRI using 3 different LGE imaging methods: conventional breath-held 2D bright-blood LGE, free-breathing 3D dark-blood LGE,^19^ and 2D dark-blood LGE reconstructed from the previous free-breathing 3D method.^12^ The authors found that both beginner (with 2 years of experience) and expert (with >10 years of experience) readers were more confident with respect to ischemic scar detection using the reconstructed 2D dark-blood LGE method compared with 2D bright-blood LGE, although statistical significance was only reached for the beginner reader. These findings are similar to the present study, where level 2 readers were significantly more confident when using dark-blood LGE compared with bright-blood LGE, while there was no significant difference for level 3 readers. The main differences with the current study, however, are the well-defined and extended experience levels according to internationally established standards, multiple readers for each experience level (3 each), and readers from 4 different international centers. Additionally, in the study of Nies et al,^12^ differences in confidence scores between the beginner and expert reader were not statistically tested.

In 2018, Villa et al^16^ conducted a study, using the same EACVI classification of competency, that investigated the effect of reader training and experience on the diagnostic accuracy of stress perfusion cardiac MRI for identification of coronary artery disease. This study found a significant effect of the level of training and experience of readers on the diagnostic accuracy, with level 1 scoring the lowest and level 3 readers scoring the highest. Since stress perfusion MRI is considered a more proficiency-demanding cardiac application compared with LGE MRI, the differences among level 1, level 2, and level 3 readers (55.7%, 65.7%, and 83.6%, respectively) were greater than in the current study, while the variability within each of the 3 levels (5.3%, 4.3%, and 2.3%, respectively) was smaller. The lack of a clear distinction among the 3 levels of reader experience in the present study might explain why statistical significance was not always reached. It should be noted, though, that the ICCs showed good interreader reliability within each level.

Training and experience of clinicians reviewing cardiac MRI scans is of high importance.^11^ The present study suggests that dark-blood LGE improves the detection of MI compared with bright-blood LGE, particularly among less experienced readers. While these findings are promising, further validation is needed to assess their generalizability. In addition, future research should explore including more advanced imaging techniques, such as 3D LGE imaging,^19^,^20^ which are increasingly adopted in clinical practice.

### Limitations

This study has several limitations that need to be discussed. First, considering that the expert serving as the reference standard was an EACVI level 3 consultant, it was expected that all level 3 readers in this study would achieve scores close to 100%. However, it is important to note that this expert had access to all available MRI sequences and clinical information, whereas the readers were provided only with dark-blood LGE or bright-blood LGE images, and the cine images. The availability of additional clinical information could influence the final conclusion concerning the presence of infarction, potentially explaining the lower-than-expected performance.

Second, assessing expertise objectively is not trivial and has specific shortcomings. For instance, EACVI levels 1–3 are primarily based on the number of reviewed cardiac MRI cases and CME hours, which may indirectly reflect expertise but do not account for factors such as innate ability and clinical judgment, especially in complex cases. Given the relatively small differences between the 3 levels, the substantial within-level variability raises concerns about the accuracy of reader classifications. Despite these challenges, the authors believe that classifying readers by expertise levels (level 1, level 2, and level 3) remains a reasonable approach, though more uniform guidelines for assessing expertise would be beneficial for future studies.

Third, among the 100 patients, 3 patients had 2 separate infarctions that were considered as 1 infarction: 2 patients had 1 large and 1 small infarction (both classified as 1 large infarction), and 1 patient had 2 small infarctions (classified as 1 small infarction). Although our statistical analysis was not designed for multiple infarctions per patient, the authors feel that this limitation, given the small numbers, would not affect the results significantly.

Lastly, during all MRI scans in this study, dark-blood LGE (performed at approximately 10 minutes postcontrast) preceded bright-blood LGE (performed at approximately 20 minutes postcontrast). Previous research has demonstrated that, as contrast agents wash out over time, T_1_ values of scar and blood diverge, enhancing scar-to-blood contrast in LGE imaging.^13^,^21^ This suggests a potential advantage for conventional bright-blood LGE over dark-blood LGE in this study. However, in clinical practice, bright-blood LGE is typically performed at 10 minutes postcontrast, which therefore may result in greater differences in the detection of MIs between dark-blood LGE and bright-blood LGE than observed in the current study.

## CONCLUSIONS

This study showed that readily available dark-blood LGE can benefit less experienced readers in correctly identifying and assessing (small) MIs compared with conventional bright-blood LGE. In addition, the use of dark-blood LGE led to increased confidence when assessing the presence and transmurality of infarctions, regardless of the level of training and experience of the readers.

## Supplementary Material

**Figure:**
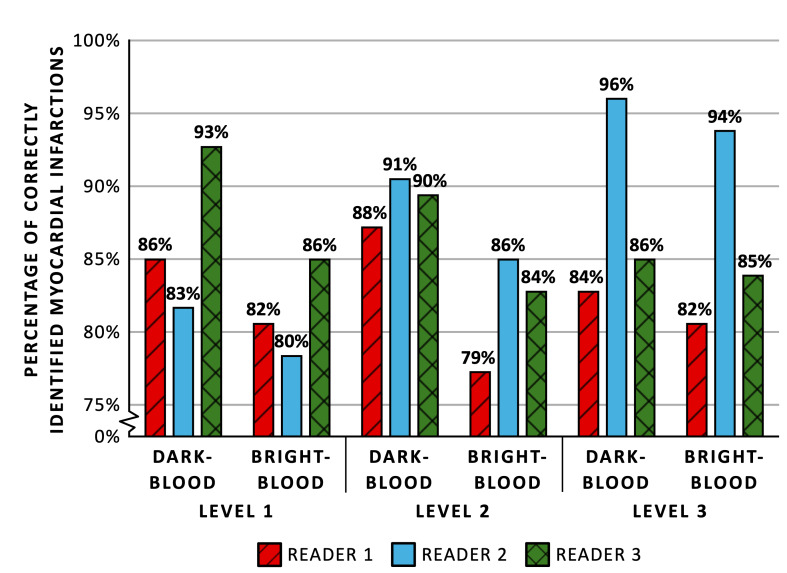


## References

[1] Safiri S Karamzad N Singh K . Burden of ischemic heart disease and its attributable risk factors in 204 countries and territories, 1990–2019. Eur J Prev Cardiol. 2022;29:420–431. 34922374 10.1093/eurjpc/zwab213

[2] Global Health Estimates: Life expectancy and leading causes of dealth and disability [WHO Website] . 2020. Available at: https://www.who.int/data/gho/data/themes/mortality-and-global-health-estimates. Accessed April 19, 2024.

[3] Roth GA Mensah GA Johnson CO . Global burden of cardiovascular diseases and risk factors, 1990–2019: update from the GBD 2019 study. J Am Coll Cardiol. 2020;76:2982–3021. 33309175 10.1016/j.jacc.2020.11.010 PMC7755038

[4] Shu T Tang M He B . Assessing global, regional, and national time trends and associated risk factors of the mortality in ischemic heart disease through global burden of disease 2019 study: population-based study. JMIR Public Health Surveill. 2024;10:e46821. 38265846 10.2196/46821 PMC10851120

[5] Kramer CM Barkhausen J Bucciarelli-Ducci C . Standardized cardiovascular magnetic resonance imaging (CMR) protocols: 2020 update. J Cardiovasc Magn Reson. 2020;22:17. 32089132 10.1186/s12968-020-00607-1 PMC7038611

[6] Jernberg T Hasvold P Henriksson M . Cardiovascular risk in post-myocardial infarction patients: nationwide real world data demonstrate the importance of a long-term perspective. Eur Heart J. 2015;36:1163–1170. 25586123 10.1093/eurheartj/ehu505

[7] Kwong RY Chan AK Brown KA . Impact of unrecognized myocardial scar detected by cardiac magnetic resonance imaging on event-free survival in patients presenting with signs or symptoms of coronary artery disease. Circulation. 2006;113:2733–2743. 16754804 10.1161/CIRCULATIONAHA.105.570648

[8] Nijveldt R Beek AM Hirsch A . Functional recovery after acute myocardial infarction: comparison between angiography, electrocardiography, and cardiovascular magnetic resonance measures of microvascular injury. J Am Coll Cardiol. 2008;52:181–189. 18617066 10.1016/j.jacc.2008.04.006

[9] Holtackers RJ Chiribiri A Schneider T . Dark-blood late gadolinium enhancement without additional magnetization preparation. J Cardiovasc Magn Reson. 2017;19:64. 28835250 10.1186/s12968-017-0372-4 PMC5568308

[10] Holtackers RJ Van De Heyning CM Chiribiri A . Dark-blood late gadolinium enhancement cardiovascular magnetic resonance for improved detection of subendocardial scar: a review of current techniques. J Cardiovasc Magn Reson. 2021;23:96. 34289866 10.1186/s12968-021-00777-6 PMC8296731

[11] Schulz-Menger J Bluemke DA Bremerich J . Standardized image interpretation and post processing in cardiovascular magnetic resonance: Society for Cardiovascular Magnetic Resonance (SCMR) board of trustees task force on standardized post processing. J Cardiovasc Magn Reson. 2013;15:35. 23634753 10.1186/1532-429X-15-35 PMC3695769

[12] Nies HMJM Martens B Gommers S . Myocardial scar detection using high-resolution free-breathing 3D dark-blood and standard breath-holding 2D bright-blood late gadolinium enhancement MRI: a comparison of observer confidence. Top Magn Reson Imaging. 2023;32:27–32. 37058709 10.1097/RMR.0000000000000304 PMC10510822

[13] Holtackers RJ Van De Heyning CM Nazir MS . Clinical value of dark-blood late gadolinium enhancement cardiovascular magnetic resonance without additional magnetization preparation. J Cardiovasc Magn Reson. 2019;21:44. 31352900 10.1186/s12968-019-0556-1 PMC6661833

[14] Krittayaphong R Zhang S Tanapibunpon P . Dark-blood late gadolinium-enhancement cardiac magnetic resonance imaging for myocardial scar detection based on simplified timing scheme: single-center experience in patients with suspected coronary artery disease. Quant Imaging Med Surg. 2022;12:1037–1050. 35111603 10.21037/qims-21-704 PMC8739141

[15] Pein S Schulz-Menger J Almeida A . Training and accreditation in cardiovascular magnetic resonance in Europe: a position statement of the working group on cardiovascular magnetic resonance of the European Society of Cardiology. Eur Heart J. 2011;32:793–798. 21289043 10.1093/eurheartj/ehq474

[16] Villa ADM Corsinovi L Ntalas I . Importance of operator training and rest perfusion on the diagnostic accuracy of stress perfusion cardiovascular magnetic resonance. J Cardiovasc Magn Reson. 2018;20:74. 30454074 10.1186/s12968-018-0493-4 PMC6245890

[17] Cerqueira MD Weissman NJ Dilsizian V . Standardized myocardial segmentation and nomenclature for tomographic imaging of the heart. A statement for healthcare professionals from the cardiac imaging Committee of the Council on clinical cardiology of the American Heart Association. Circulation. 2002;105:539–542. 11815441 10.1161/hc0402.102975

[18] Kim YJ Kim RJ . The role of cardiac MR in new-onset heart failure. Curr Cardiol Rep. 2011;13:185–193. 21399925 10.1007/s11886-011-0179-0

[19] Holtackers RJ Gommers S Van De Heyning CM . Steadily increasing inversion time improves blood suppression for free-breathing 3D late gadolinium enhancement MRI with optimized dark-blood contrast. Invest Radiol. 2021;56:335–340. 33273374 10.1097/RLI.0000000000000747

[20] Polacin M Gastl M Kapos I . Novel magnetic resonance late gadolinium enhancement with fixed short inversion time in ischemic myocardial scars. Invest Radiol. 2020;55:445–450. 32459683 10.1097/RLI.0000000000000655

[21] Kellman P Arai AE McVeigh ER . Phase-sensitive inversion recovery for detecting myocardial infarction using gadolinium-delayed hyperenhancement. Magn Reson Med. 2002;47:372–383. 11810682 10.1002/mrm.10051 PMC2041905

